# Conductivity of PEDOT:PSS on Spin-Coated and Drop Cast Nanofibrillar Cellulose Thin Films

**DOI:** 10.1186/s11671-015-1093-y

**Published:** 2015-10-05

**Authors:** Dimitar Valtakari, Jun Liu, Vinay Kumar, Chunlin Xu, Martti Toivakka, Jarkko J. Saarinen

**Affiliations:** Laboratory of Paper Coating and Converting, Center for Functional Materials (FunMat), Abo Akademi University, Porthansgatan 3, 20500 Åbo/Turku, Finland; Laboratory of Wood and Paper Chemistry, Abo Akademi University, Porthansgatan 3, 20500 Åbo/Turku, Finland

**Keywords:** PEDOT:PSS, Nanofibrillar cellulose (NFC), Conductivity, Thin films, 61.46.-w (structure of nanoscale materials), 68.37.-d (microscopy of surfaces, interfaces, and thin films), 81.05.Lg (polymers and plastics), 81.07.-b (nanoscale materials and structures: fabrication and characterization)

## Abstract

Aqueous dispersion of conductive polymer poly(3,4-ethylenedioxythiophene)–poly(styrenesulfonate) (PEDOT:PSS) was deposited on spin-coated and drop cast nanofibrillar cellulose (NFC)–glycerol (G) matrix on a glass substrate. A thin glycerol film was utilized on plasma-treated glass substrate to provide adequate adhesion for the NFC-glycerol (NFC-G) film. The effects of annealing temperature, the coating method of NFC-G, and the coating time intervals on the electrical performance of the PEDOT:PSS were characterized. PEDOT:PSS on drop cast NFC-G resulted in 3 orders of magnitude increase in the electrical conductivity compared to reference PEDOT:PSS film on a reference glass substrate, whereas the optical transmission was only slightly decreased. The results point out the importance of the interaction between the PEDOT:PSS and the NFC-G for the electrical and barrier properties for thin film electronics applications.

## Background

Thin, lightweight, and flexible conductive films are useful for many applications from intelligent packaging, solar panels, radio frequency identification (RFID) tags to medical implants, wearable computers, and various sensors [1]. Traditionally, such films have been produced on plastics, glass, and other nonrenewable materials. However, the recent environmental concerns have resulted in the search for alternative and more sustainable materials. These must be cost-effective, widely available, and both ecologically and economically sustainable.

Cellulose is the most abundant biopolymer on the Earth and thus a suitable candidate for replacing fossil fuel-based solutions. Within the past decade, nanostructured cellulose-based materials have raised large attention due to their unique properties. Nanofibrillar cellulose (NFC) has a high aspect ratio, large surface area, and high strength [2]. NFC can be utilized in various end-use products ranging from thin films, coatings, and composites to aerogels and hydrogels. Recently, NFC-based conductive films and composites for electronics applications have been studied; see, e.g., reviews [3, 4]. NFC films have good thermal [5–7] and chemical stability [8], tunable optical properties [5, 6, 9], high toughness [10], and low surface roughness [11]. Therefore, NFC films can be used as a substrate for flexible and transparent electronic devices for cost-effective manufacturing in a roll-to-roll process flow.

Various strategies have been employed to induce conductivity in NFC films. The NFC film can be coated [5, 8, 9, 12] or printed [9, 11, 13] with a conductive material. Alternatively, the conductive material can be intermixed with the NFC fibers that can be cast into a conductive composite film [6, 10, 14–17]. The conductive material can also be directly incorporated onto the NFC fibers using, for example, in situ chemical polymerization [18] or carbonization [19] that allows conductive films to be produced from such conductive fibers [20]. Different conductive materials such as silver nanowires [9, 12] or nanoparticles [11, 13], tin-doped indium oxide [9], carbon nanotubes (CNTs) [5, 8–10, 12, 14, 15, 17, 20, 21], graphene oxide [19], ZnSe quantum dots [6], polypyrrole [18], polyaniline doped with camphorsulphonic acid [16], poly(p-phenylene ethynylene) [16] and poly(3,4-ethylenedioxythiophene)–poly(styrenesulfonate) (PEDOT:PSS) [5, 9, 20, 21] have been used in the literature. Nanostructured cellulose-based electronics have been studied, e.g., in flexible supercapacitors [22], flexible nanopaper transistors with high transmission and low surface roughness [23], photovoltaic cells based on cellulose nanocrystal substrates [24], nonvolatile memory based on cellulose nanofiber paper [25], subcomponents made from nanofibrillar cellulose for “cut, stick and peel”-based flexible electronics [26], and recently cellulose nanofibril paper-based high-performance flexible electronics [27].

PEDOT:PSS is a solution-processable conductive polymer that offers flexibility, high transparency, high thermal stability, low production cost, and compatibility with aqueous solution-based deposition techniques. PEDOT:PSS has a lower electrical conductivity than the other conductive polymers or metal oxides. However, the conductivity can be improved by addition of polyalcohols such as glycerol [28], by adding polyelectrolytes [29], or by solvent [30] or an acid treatment [31, 32]. Highly conductive and transparent PEDOT:PSS films have been studied, e.g., for replacing indium tin oxide (ITO) in photovoltaics [33, 34] and touch screens [35]. Recently, a solar cell [9] and an OLED device [5] were demonstrated using a spin-coated conductive PEDOT:PSS on NFC substrate. Additionally, PEDOT:PSS has been used with CNTs in the layer-by-layer coating of wood microfibers for paper-based batteries [20] and capacitors [21].

In this work, we report a simple fabrication method of conductive PEDOT:PSS films on spin-coated or drop cast NFC-glycerol (NFC-G) layer on top of an oxygen plasma-activated glass substrate. We use glycerol as an anchor layer between the NFC-G layer and glass substrate since the NFC-G solution cannot be coated directly onto a pure plasma-activated glass substrate. A glycerol anchor layer provides good adhesion and reproducible results that allows a systematic study of the interaction between PEDOT:PSS and NFC-G coating. The electrical properties are studied as a function of the annealing temperature, the spin coating and drop casting of NFC, and the coating time intervals during film deposition. The effect of glycerol as a plasticizer on film formation and the resulting conductivity is discussed as well. PEDOT:PSS on NFC can be used to produce humidity or amine sensors, which can find applications in food packaging industry. These films can also be utilized in solar cell applications due to their tunable optical properties.

## Methods

The NFC suspension was prepared from bleached birch Kraft pulp using 2,2,6,6-tetramethylpiperidine-1-oxyl (TEMPO)-mediated oxidation followed by mechanical disintegration as reported by Liu et al*.* [36]. Ten grams of the pulp fibers were dispersed in 600 mL deionized (DI) water (Millipore 18.2 MΩ). The TEMPO (0.1 mmol/g fiber) and NaBr (1.0 mmol/g fiber) were dissolved in 300 mL DI water and then mixed with the pulp slurry. The concentration and the pH of the pulp were adjusted to 0.1 % and 10.0, respectively. The oxidation was started by adding the NaClO (10 mmol/g fiber) solution dropwise. All NaClO solution was added for 8 h. During the reaction, the pH of the reaction mixture was kept at 10.5 by adding 0.5 M NaOH. After 24.0-h reaction, the mixture was precipitated in ethanol and purified by washing with DI water and centrifugation at 3500 rpm for 10 min for three times. The oxidized fibers were diluted to a concentration of 0.5 % and mechanically fibrillated with a domestic blender (OBH Nordica 6658, Denmark) for 5 min. The carboxylate content (1.79 ± 0.11 mmol/g) of the resulting NFC was determined by conductometric titration [36]. The chemicals were purchased from Sigma Aldrich and used without further purification.

The NFC and glycerol (≥99.0 %, Sigma-Aldrich, St. Louis, USA) solutions were prepared by weighing and diluting to given strengths using DI water and small 1.5 mL microcentrifuge tubes. All NFC-G coatings were well mixed and applied fresh on the surface immediately after the mixture preparation.

All samples were prepared on washed, cleaned, and dried clear microscope slide glasses cut into the size of 2.5 × 2.5 × 0.1 cm^3^. Furthermore, the glass slide surfaces were oxygen plasma treated in the etch mode in a high vacuum sample sputter coater (SCD 050, Bal-Tec AG, Balzers, Liechtenstein; now Leica Microsystems GmbH., Wetzlar, Germany) for 60 s at 20 mA and 0.05 mbar vacuum pressure for 3–15 min prior to spin coating. The plasma treatment lowered the water contact angle (KSV Cam 2000, Biolin Scientific Inc., Espoo, Finland) on the glass surface from 14° to 0°.

**Fig. 1:**
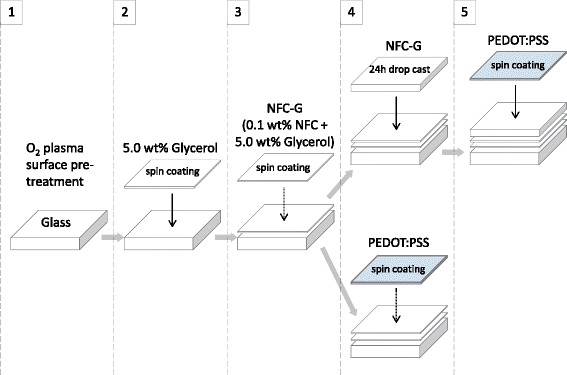
The NFC-G sample preparation steps. The glass slide surface is treated by oxygen plasma (*1*) followed by spin coating of 5.0 wt% glycerol anchor layer (*2*), NFC-G spin coating (*3*), and PEDOT:PSS spin coating (*4*). The drop cast NFC-G samples were prepared by an additional step before the PEDOT:PSS spin coating

Figure 1 shows the two different approaches for the sample preparation with spin-coated and drop cast NFC-G mixture. The samples were spin coated (KW-4A, Chemat Technology Inc., USA) at different speeds depending on the material: 5.0 wt% glycerol (the anchor layer) at 1000 rpm for 60 s, NFC-G mixtures at 1000 rpm for 60 s and homogenized standard PEDOT:PSS (Clevios PH 500, Heraeus Holding GmbH, Germany) at 1500 rpm for 60 s. The additional drop cast NFC-G coatings on precoated NFC-G layer were prepared out of 200 μL NFC-G solution of a given strength and mixture ratio covering the substrate area completely from edge to edge and then left to dry for 24 h at room temperature (RT) of 24 °C and relative humidity (RH) of 50 %. All samples were left to dry for 15 to 60 min in between the coating steps, and no wet-on-wet coatings were applied at any stage of this work.

The spin-coated NFC-G layer serves two purposes as shown in Fig. 1: first, the PEDOT:PSS can be directly spin coated on top of it, and secondly, it serves as a rigid underlayer in which the drop cast NFC-G coating adheres preventing withdrawal from the edge during drying. We wish to emphasize that NFC or NFC-G did neither adhere to the plain glass slide surface nor did it form a clean, uniform coating on the oxygen plasma-treated glass surface. However, addition of a 5.0 wt% glycerol coating as an anchor layer after the oxygen plasma surface treatment step resulted in good-quality spin-coated NFC-G films. The adhesion of the glycerol anchor layer was better on the oxygen plasma-coated surfaces than on an untreated plain glass. The glycerol coating has several advantages as it is colorless and semitransparent making it suitable for transmission measurements. It is also a plasticizer and a humectant for the NFC films to avoid brittleness and a well-known secondary dopant that can increase the overall electrical conductivity of the PEDOT:PSS by 2 to 3 orders of magnitude.

PEDOT:PSS samples were spin coated and annealed for 20 min at a given temperature range from 60 to 130 °C (Model 200, Memmert GmbH, Schwabach, Germany). Samples were kept aside prior to annealing for 20 to 30 min. Samples were stored overnight in dark and in humidity-controlled conditions at RH 50 % and measured for electrical conductivity on the following day. The sheet resistance was measured over two parallel hand-painted conductive silver paint (Electrolube) stripes with inner borders 10 mm apart. A Keithley 2100 Digital Multimeter was used for sheet resistance measurements at 24 °C and RH 50 %. The scanning electron microscope (SEM, LEO 1530 VP Gemini, Carl Zeiss Microscopy Gmbh, Oberkochen, DE) was used to image the thickness and structure of the PEDOT:PSS-coated NFC-G samples on glass. These were either freeze-fractured after dipping in liquid N_2_ for a minimum of 5 s or alternatively fractured under ambient conditions. The sample light transmission was measured using a Perkin Elmer Lamba 900 spectrophotometer with an integrating sphere setup and the UV Winlab software.

## Results and Discussion

### The Effect of Annealing Temperature and NFC-G Mixture on the PEDOT:PSS Sheet Resistance

**Fig. 2:**
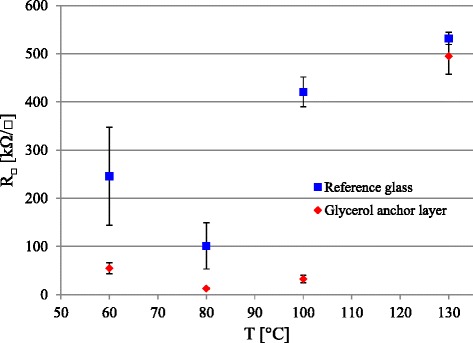
PEDOT:PSS sheet resistance on reference glass and glass with glycerol anchor layer

PEDOT:PSS aqueous dispersion coatings are typically annealed to increase their conductivity. Figure 2 shows the effect of annealing temperature on conductivity of the spin-coated PEDOT:PSS deposited on oxygen plasma-treated reference and glycerol-treated glass. The used annealing temperatures vary from 60 to 130 °C that are below the recommended drying temperature given by the polymer manufacturer of 130 °C. The PEDOT:PSS films become more stable above 80 °C, and the scatter in the measurement data decreases with the raising annealing temperature. On a pure glass substrate, the PEDOT:PSS reaches a sheet resistance minimum at the 80 °C annealing temperature.

The spin-coated PEDOT:PSS on glycerol anchor layer resulted in a significant drop in the sheet resistance values at lower annealing temperatures from 60 to 100 °C range. This is expected as glycerol is traditionally considered as a secondary dopant for the PEDOT:PSS. It blends with the PEDOT:PSS and improves the conductivity by allowing the PEDOT and the PSS components to restructure morphologically. The PEDOT:PSS conductivity can also be improved by blending, e.g., some polyols into the PEDOT:PSS that evaporate or pass through the PEDOT:PSS layer without a trace. On the glass substrates, the lowest sheet resistances are found in the middle and lower end of the used annealing temperature range.

**Fig. 3:**
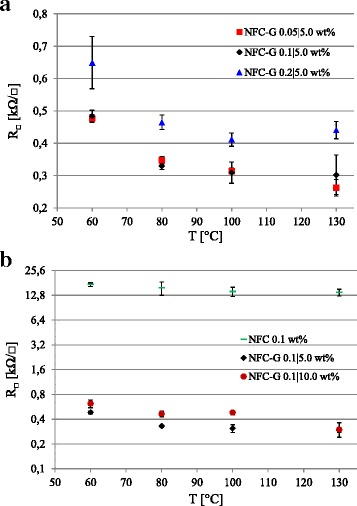
PEDOT:PSS sheet resistance on drop cast NFC-G samples with **a** variable NFC concentration at a constant glycerol concentration of 5.0 wt% and **b** constant NFC concentration of 0.1 wt% with variable glycerol concentration

The PEDOT:PSS coatings on drop cast NFC-G with a varying NFC concentration at 5.0 wt% glycerol in Fig. 3a show a significant decrease in the sheet resistance by 3 orders of magnitude compared to the reference PEDOT:PSS on glass without NFC. The drop cast NFC-G coatings were produced with three different NFC strengths, i.e., with three different viscosities while keeping the glycerol plasticizer level constant (5.0 wt%) in the NFC-G solution mixture. The 0.05 and 0.1 wt% NFC concentrations gave almost identical results. The sheet resistances dropped and leveled out with an increasing annealing temperature. The gel-like blend with the highest viscosity of 0.2 wt% NFC had the highest sheet resistance. This can be caused by the reduced deformability of NFC due to the high viscosity, which can result in lowered absorption and blending at the NFC/PEDOT:PSS interface. In the reference PEDOT:PSS, solvent evaporation takes place from a thin layer whereas NFC and glycerol absorb water from the aqueous PEDOT:PSS solution resulting in a different drying mechanism that is susceptible for cracking. Therefore, on higher NFC concentrations, uneven shrinkage and mechanical stress during the annealing can result in cracking and reduced PEDOT:PSS film quality, and thus, a higher sheet resistance as observed in Fig. 3a. However, the sheet resistance of PEDOT:PSS on drop cast NFC-G is significantly lower than the PEDOT:PSS reference.

Figure 3b shows the effect of glycerol concentration on the sheet resistance of PEDOT:PSS at a constant NFC concentration of 0.1 wt%. The NFC itself is sufficient to reduce the reference PEDOT:PSS sheet resistance levels by a factor of 30 from 550 kΩ/□ down to <18 kΩ/□. This may be due to water still present in the NFC-G layer that can allow vapor transmission through and rearrangement of the PEDOT:PSS layer [37, 38]. The lowest sheet resistance is observed with the 5.0 wt% glycerol concentration, which is expected as glycerol is a secondary dopant for the PEDOT:PSS. However, a further increase of glycerol content to 10.0 wt% does not improve conductivity as the mechanical properties of the drop cast NFC-G layers start to fail and disintegrate during the spin coating. The overall PEDOT:PSS stability improves towards higher temperatures without the sheet resistance increase as observed in Fig. 2. We conclude that the pure drop cast NFC coating improves the PEDOT:PSS conductivity by an order of magnitude. However, a much larger improvement is achieved through the combination of glycerol and water originating from the NFC-G mixture that allows vapor transmission and rearrangement of the PEDOT:PSS coating.

### Sheet Resistance of PEDOT:PSS on Spin-Coated NFC-G

Drop casting is a slow process whereas spin coating provides a fast track for prototyping. In our study, spin coating of NFC-G films reduced the processing time from 24 h down to a few minutes. In addition, the sample to sample variation was reduced with spin coating. This is especially important with multilayer structures as every additional sample preparation step with successive coating layers can be a source of error.

**Fig. 4:**
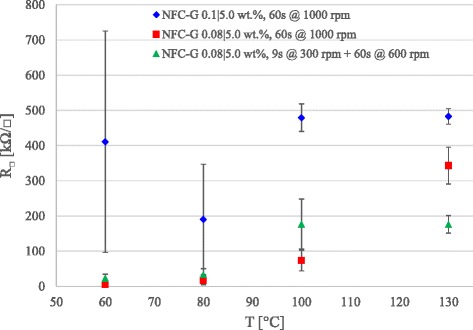
PEDOT:PSS sheet resistance on spin coated NFC-G samples with variable NFC concentration and spin coating parameters at a constant glycerol concentration of 5.0 wt%

Figure 4 shows the PEDOT:PSS sheet resistances on a spin-coated NFC-G. We observe that PEDOT:PSS on 0.1/5.0 wt% NFC-G behaves similarly to the reference PEDOT:PSS spin-coated directly on a glass as shown in Fig. 2. The large standard deviations in the 60 and 80 °C points originate from the PEDOT:PSS instability that is absent in the 100 and 130 °C. Similar results indicate that the PEDOT:PSS and the NFC-G coatings act as separate, isolate entities with no interaction between the two layers during the spin coating or annealing.

A reduction in NFC concentration from 0.1 wt% down to 0.08 wt% produces an NFC-G mixture with a lower viscosity. Thus, at a constant spinning rate (1000 rpm), a thinner film will be produced. The sheet resistance of the PEDOT:PSS is reduced due to the PEDOT:PSS interaction with the glycerol at 60 and 80 °C. Furthermore, a slow initialization rate at 300 rpm will retain more liquid on the substrate that results in a thicker film. At lower temperatures, the sheet resistances remain at the previous levels whereas at 100 and 130 °C, the performance is stabilized approximately to 200 kΩ/□ that is significantly lower compared to the PEDOT:PSS reference (550 kΩ/□).

We can conclude that the higher NFC concentration of 0.1 wt% forms a tight and dense layer with sufficient barrier properties to prevent the glycerol interaction with the PEDOT:PSS. On the other hand, at a lower NFC concentration of 0.08 wt%, the NFC-G layer is less dense allowing the glycerol to migrate into the PEDOT:PSS layer during the spin coating and annealing. The 100 °C point suggests that the NFC component dries quicker and becomes restrictive, blocking the glycerol and water from the PEDOT:PSS. Hence, the spin-coated NFC-G films can be customized to act as either a barrier (0.1 wt% NFC) or as a regulator (0.08 wt% NFC) that controls the release of glycerol and water into the PEDOT:PSS layer.

**Fig. 5:**
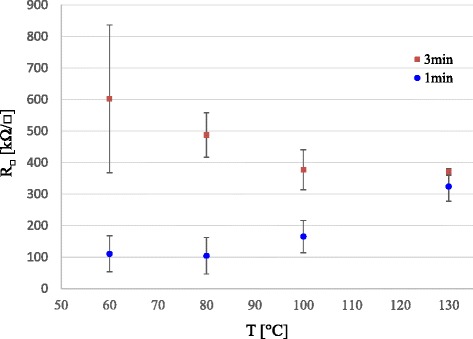
PEDOT:PSS sheet resistance on spin coated NFC-G samples with variable time interval between the spin coating and annealing

Finally, the time dependence of the start of the annealing from the PEDOT:PSS coating is shown in Fig. 5. It is clearly seen that starting the annealing instantly 1 min after the coating results in lower sheet resistance with lower annealing temperatures than the 3-min interval. The 1-min samples have higher moisture content, and the samples annealed at higher temperatures may drive the water and perhaps some glycerol rapidly out from the PEDOT:PSS/NFC-G interface region, disallowing PEDOT and PSS to rearrange. The sheet resistance levels in Fig. 5 at 80 °C are approximately twice as high to those in Fig. 4. A closer look reveals that the 3-min samples have a similar instability issue at 60 °C as with the PEDOT:PSS reference response shown in Fig. 2. The overall performance improves and becomes more stable converging to 300–400 kΩ/□ sheet resistance level between annealing temperatures of 80 and 130 °C.

### Coating Thickness, Wetting Characteristics, and Optical Transmission

An objective with our NFC-G model system on glass substrate was to retain the same semitransparent character provided by unsupported, standalone NFC films. The benefit of our model system is that the flat NFC-G films supported on the glass are suitable for the PEDOT:PSS spin coating. Our conductivity results in Figs. 2, 3, 4, and 5 show that these samples are reproducible and thus suitable both for electrical and optical characterization.

The sample thicknesses of the PEDOT:PSS-coated NFC-G layers were determined using SEM. The samples were either annealed at 130 °C or left as such with no annealing. The annealing step is essential for the conductivity, electrical stability, and smoothness of the PEDOT:PSS layer. Annealing causes shrinkage of the PEDOT:PSS liquid dispersion particle, vertical segregation into a mainly PEDOT phase at the bottom, and a PSS phase on top as well as degradation over a prolonged exposure to heat [39].

**Fig. 6:**
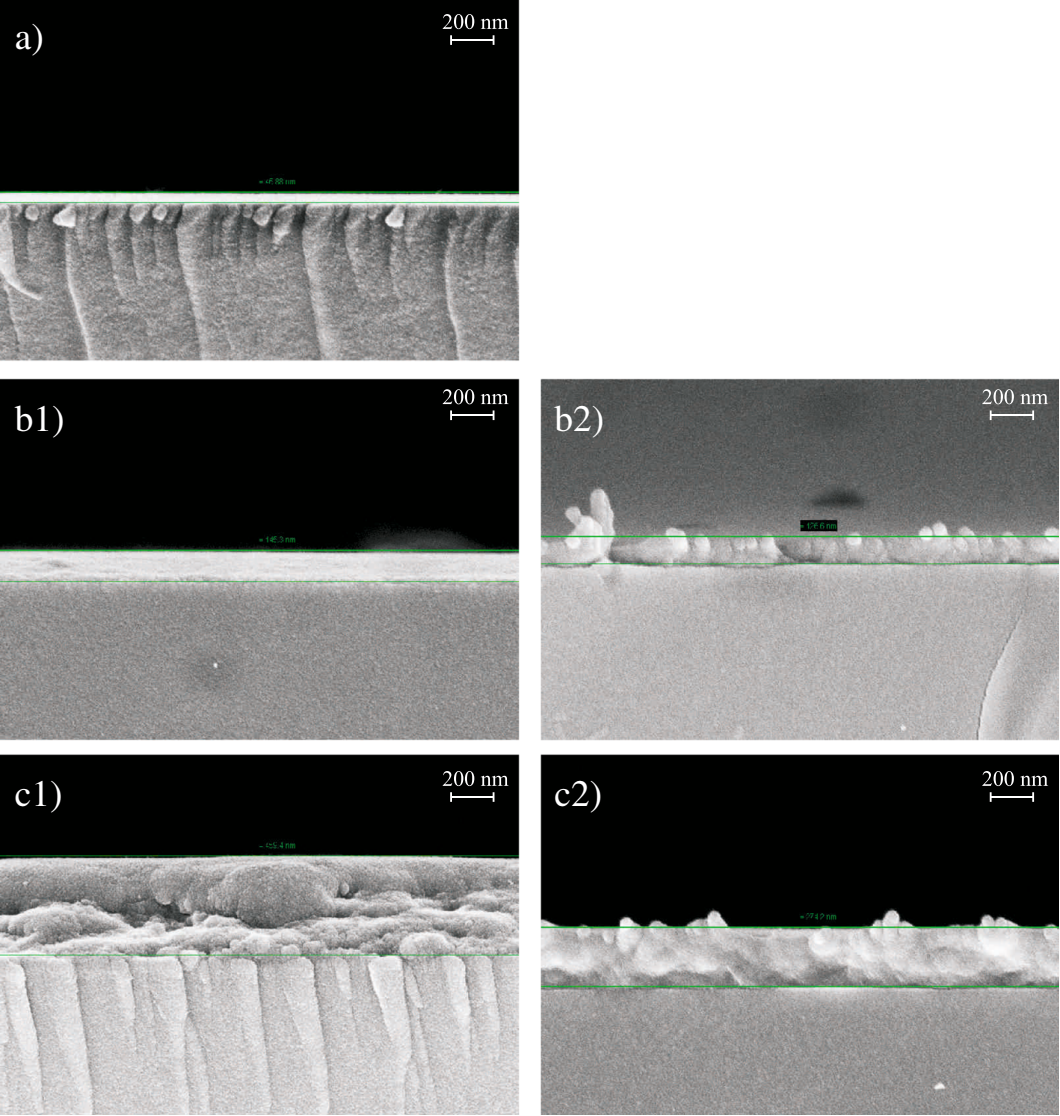
Left hand column, annealed (130 °C) and freeze fractured samples: (**a**) PEDOT:PSS reference, thickness 47 nm; (**b1**) PEDOT:PSS on spin-coated NFC-G, thickness 138–145 nm; (**c1**) PEDOT:PSS on drop cast NFC-G, thickness 438–458 nm. Right hand column samples without annealing and freeze fracturing: (**b2**) PEDOT:PSS on spin-coated NFC-G, thickness 127 nm; (**c2**) PEDOT:PSS on drop cast NFC-G, thickness 274 nm

Figure 6 shows cross sections of the PEDOT:PSS samples with and without annealing together with the spin-coated reference PEDOT:PSS on the oxygen plasma-activated glass on top (a). The annealed samples are shown to the left in Fig. 6 with spin coating (b1) and drop casting (c1) and the corresponding samples without annealing to the right, spin coated (b2), and drop cast (c2). The spin coating causes film shrinkage due to compression and fast evaporation of water from the thin films. Furthermore, annealing causes shrinkage due to drying. The cross section samples for the SEM imaging are commonly freeze fractured in liquid nitrogen. Unfortunately, moisture absorbed from the surrounding air can cause sample swelling with hygroscopic materials. In Fig. 6, on the left (a1, b1, c1), all drying steps are present, whereas on the right (b2, c2), the drying steps were reduced to a minimum. Finally, the cross section samples to the right were fractured at room temperature without freezing.

The spin-coated PEDOT:PSS sample without annealing (b2) has a thickness of 127 nm. This is slightly less compared to the 138–145 nm of the annealed and freeze-fractured counterpart (b1). This suggests that the spin-coated NFC-G has a dense texture being fairly resistant to the moisture. This is also supported by the water contact angle results. The spherical PEDOT:PSS water dispersion particles can be distinguished on the surface in the sample (b2). This suggests that the spin-coated NFC-G layer accounts for approximately 80 nm and the PEDOT:PSS resting on top the remaining 47 nm from the total thickness of 127 nm.

The drop cast PEDOT:PSS samples have a significantly thicker NFC-G layer than the spin-coated counterparts as drop-casting produces a less dense texture with high water release and uptake ability. The drop cast PEDOT:PSS-coated sample shrank during annealing. However, the sample swells after the freeze fracturing to 438–485 nm as seen in the sample (c1). Of the total thickness of 274 nm in the sample (c2) without annealing, approximately 230 nm belongs to the NFC component. The water contact angle results show that the PEDOT:PSS coating on top provides a barrier to moisture. Therefore, it is reasonable to assume that the swelling is mainly caused by moisture entering from the open face of the fractured side, not from the top.

**Fig. 7:**
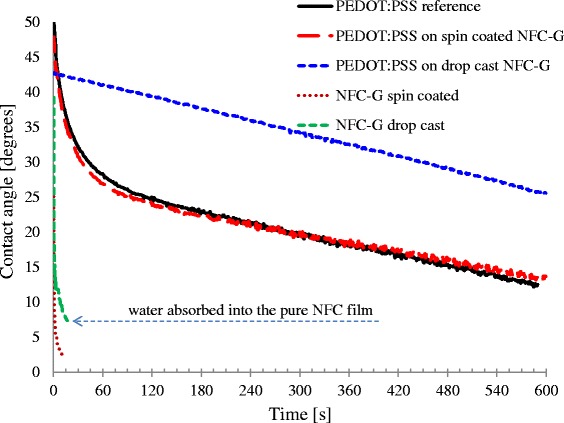
Water contact angles using the sessile drop technique with a 4-μL drop volume

Water contact angle measurements are shown in Fig. 7. The PEDOT:PSS-coated samples were annealed at 130 °C, and all the samples were stored at RH 50 % and measured 24 h after sample preparation was completed. The spin-coated and drop cast pure NFC samples are highly hydrophilic with the latter showing very strong absorption of water between the 15 and 40-s time interval causing swelling and deformation of the NFC layer. Some marks from the drying water droplets were observed on the surface of the spin-coated NFC after completed drying. This indicates a limited water absorption into the NFC layer. We also tested the water contact angles on glycerol film, and the results were visually identical to the spin-coated NFC except the strong uneven and noncircular wetting pattern outwards from the wetting center.

The PEDOT:PSS films were less hydrophilic in comparison to the uncoated NFC-G layers. The spin-coated PEDOT:PSS on NFC-G behaved identically to the PEDOT:PSS reference on glass. This indicates that the sessile drop method is insensitive to the morphological changes that can take place in the spin-coated PEDOT:PSS on NFC-G resulting in a better conductivity. The PEDOT:PSS on drop cast NFC-G shows approximately 15° higher water contact angles than the reference PEDOT:PSS. In general, the annealing step has a smoothening effect on the PEDOT:PSS films. However, this may be compromised by the pores and pinholes in the NFC-G layer caused by water and glycerol evaporation through the PEDOT:PSS film. Nevertheless, the barrier properties of the PEDOT:PSS coating are quite remarkable as the water contact angle drops monotonously, at the same rate as the reference, suggesting only a limited water absorption. Visual inspection of the samples revealed only light marks on the surface from the drying water droplet.

**Fig. 8:**
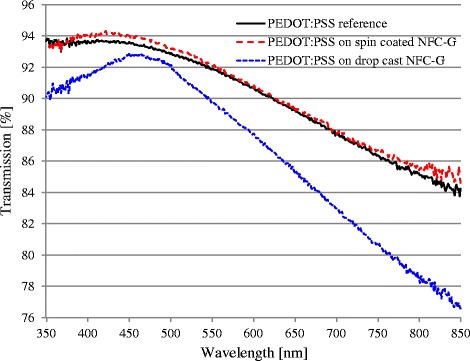
Optical transmission of PEDOT:PSS on reference glass, spin-coated NFC-G, and drop cast NFC-G coatings annealed at 130 °C

The optical transmission results are in Fig. 8 for the spin-coated PEDOT:PSS on reference glass and on spin-coated and drop cast NFC-G. The spin-coated sample data overlaps with that of the reference with transmission varying from approximately 94 to 84 % for the wavelength range 350–850 nm. Both maintain the same light blue color shade, and the components in the NFC-G layer seem not to affect the transmission. This is in accordance by the dense texture and strong barrier properties of the NFC-G layer observed in the SEM and the contact angle measurements. On the other hand, the PEDOT:PSS on drop cast NFC-G has 1 to 8 points lower transmission across the measured spectral range of 350–850 nm. The transmission loss is a fairly small sacrifice in comparison to the enhanced conductivity increase of three orders of magnitude. The transmission loss is caused by a small color shift towards deeper blue as a result of the PEDOT:PSS absorption into the NFC-G layer. The PEDOT:PSS liquid dispersion consists of more than 95 % water that is easily absorbed by the drop cast NFC-G layer as seen in the contact angle measurement results in Fig. 7. The same applies to the PEDOT:PSS liquid dispersion. The submicron thickness of the colorless and semitransparent NFC-G film has a minor impact on the transmission drop. Transmission results for samples annealed at temperatures from 60 to 130 °C were rather similar, and the results stayed within one percentage point for all spin-coated samples and within 3 percentage points for all drop cast samples.

## Conclusions

The conductive polymer PEDOT:PSS is typically applied on transparent surfaces such as glass or plastic film. Our work is focused on PEDOT:PSS on NFC-G, which can produce stand-alone, semi-transparent films. NFC can provide sustainable, recyclable, and biodegradable alternatives to glass and plastic substrates.

Pure NFC films are very brittle but can be converted to flexible ones by blending with a suitable plasticizer. Here, we use glycerol as a plasticizer due to its additional multifunctional characteristics: it is colorless, semitransparent, adhesive, water-soluble, and a well-known secondary dopant for the PEDOT:PSS. The semi-transparent NFC film, unlike glass and plastic, can have added functionality and be an active substrate with tailored properties that will interact and enhance the conductivity of the coated PEDOT:PSS on top of the NFC-G.

NFC films are most commonly produced through drop casting that is a time-consuming process. The dry film does not adhere to glass or plastic substrate. Gluing the NFC film onto a solid substrate may alter the optical and structural film characteristics. Therefore, an optically semi-transparent model system was developed to study the PEDOT:PSS interaction on an NFC-G layer immobilized on glass either through drop casting or spin coating. These techniques enable spin coating of PEDOT:PSS on top of the NFC-G sample combining fast prototyping with good reproducibility. The electrical conductivity of PEDOT:PSS improved 3 orders of magnitude on the drop cast NFC-G samples. The high conductivity is a combined result from the water content and the glycerol in the NFC-G film allowing annealing of the samples across a broader temperature range.

The spin-coated PEDOT:PSS on NFC-G samples provided a sensitive study platform. The conductivity levels were much lower than with drop cast NFC-G, but the changes in conductivity levels allowed a better understanding about the interaction between the PEDOT:PSS and the NFC-G. Clearly, the water content has a significant impact on the PEDOT:PSS. More importantly, the moisture level in the NFC-G film has a decisive impact on the PEDOT:PSS conductivity level when the samples are annealed. Environmental stability of the transparent, conductive PEDOT:PSS films on NFC-G substrate is a key property for practical applications that can either degrade the electrical functionality or be utilized, for example, in gas or humidity sensing of the environment. This work was carried out in well-controlled conditions to study fundamental interactions between PEDOT:PSS and NFC-G with minimized environmental variables. We plan to return on this issue in a future communication.

We believe that conductive PEDOT:PSS films on NFC-G have potential for many applications in flexible electronics and sensors in the future.
